# Design and Usability Evaluation of Mobile Voice-Added Food Reporting for Elderly People: Randomized Controlled Trial

**DOI:** 10.2196/20317

**Published:** 2020-09-28

**Authors:** Ying-Chieh Liu, Chien-Hung Chen, Yu-Sheng Lin, Hsin-Yun Chen, Denisa Irianti, Ting-Ni Jen, Jou-Yin Yeh, Sherry Yueh-Hsia Chiu

**Affiliations:** 1 Department of Industrial Design College of Management Chang Gung University Taoyuan City Taiwan; 2 Department of Internal Medicine Health Promotion Center Chang Gung Memorial Hospital Taoyuan Taiwan; 3 Cybersecurity Technology Institute Institute for Information Industry Taipei Taiwan; 4 Department of Nutrition Therapy Chang Gung Memorial Hospital Taoyuan Taiwan; 5 Department of Chemical and Materials Engineering College of Engineering Chang Gung University Taoyuan Taiwan; 6 Department of Health Care Management College of Management Chang Gung University Taoyuan Taiwan; 7 Department of Health Care Management and Healthy Aging Research Center College of Management Chang Gung University Taoyuan Taiwan; 8 Division of Hepatogastroenterology Department of Internal Medicine Kaohsiung Chang Gung Memorial Hospital Kaohsiung Taiwan

**Keywords:** voice-added design, food report, elderly, usability evaluation, automatic speech recognition, mHealth, randomized trial

## Abstract

**Background:**

Advances in voice technology have raised new possibilities for apps related to daily health maintenance. However, the usability of such technologies for older users remains unclear and requires further investigation.

**Objective:**

We designed and evaluated two innovative mobile voice-added apps for food intake reporting, namely voice-only reporting (VOR) and voice-button reporting (VBR). Each app features a unique interactive procedure for reporting food intake. With VOR, users verbally report the main contents of each dish, while VBR provides both voice and existing touch screen inputs for food intake reporting. The relative usability of the two apps was assessed through the metrics of accuracy, efficiency, and user perception.

**Methods:**

The two mobile apps were compared in a head-to-head parallel randomized trial evaluation. A group of 57 adults aged 60-90 years (12 male and 45 female participants) was recruited from a retirement community and randomized into two experimental groups, that is, VOR (n=30) and VBR (n=27) groups. Both groups were tested using the same set of 17 food items including dishes and beverages selected and allocated to present distinct breakfast, lunch, and dinner meals. All participants used a 7-inch tablet computer for the test. The resulting data were analyzed to evaluate reporting accuracy and time efficiency, and the system usability scale (SUS) was used to measure user perception.

**Results:**

For eight error types identified in the experiment, the VBR group participants were significantly (*P*<.001) more error prone owing to the required use of button-tapping actions. The highest error rates in the VOR group were related to incomprehensible reporting speech (28/420, 6.7%), while the highest error rates in the VBR group were related to failure to make required button taps (39/378, 10.3%). The VOR group required significantly (*P*<.001) less time to complete food reporting. The overall subjective reactions of the two groups based on the SUS surpassed the benchmark and were not significantly different (*P*=.20).

**Conclusions:**

Experimental results showed that VOR outperformed VBR, suggesting that voice-only food input reporting is preferable for elderly users. Voice-added apps offer a potential mechanism for the self-management of dietary intake by elderly users. Our study contributes an evidence-based evaluation of prototype design and selection under a user-centered design model. The results provide a useful reference for selecting optimal user interaction design.

**Trial Registration:**

International Standard Randomized Controlled Trial Registry ISRCTN17335889; http://www.isrctn.com/ISRCTN17335889.

## Introduction

### Background

Older people are at increased risk of malnutrition [1-3], which can increase a range of health risks [4,5]. To prevent malnutrition in seniors, self-monitoring of food intake is a critical component of macronutrient intake assessment and calorie calculation [6]. Regular screening of dietary factors from food intake can help to identify individuals at risk of malnutrition [7,8]. Advances in digital technologies are creating new options for delivering health interventions to seniors [9,10], and powerful new software and hardware tools can support improved health maintenance through the collection, analysis, and interpretation of dietary intake data [11,12]. Digital dietary intake tracking has substantial potential for improving related health or nutrition outcomes [13]. Vogels [14] found that seniors actively adopted new technologies in their daily lives, and among these, mobile health apps offer many potential advantages including accessibility, scalability, and cost-effectiveness [15,16].

### Challenges in Operation

Older adults typically experience reduced physical and physiological functioning that can increase the challenge involved in operating mobile apps, such as tapping buttons and scrolling down the screen [17], and such users often require app developers to provide enhanced interface usability to make operation faster and more accurate [16-21]. Recent technological advancements, such as voice and speech recognition [22,23], have added a new dimension to health app interface design for older adults [24]. While some studies have identified potential benefits from such technologies [25,26], there is little evidence of their effectiveness. Voice interactions are highly domain oriented. Previous research [24] stressed the need to provide evidence of effectiveness and found that, among commercial apps, diet and calorie tracking apps rarely use voice input for food intake reporting. Exploiting the potential benefits of such technologies depends on the degree to which they improve usability for the intended users. Effective interface development usually requires the active involvement of target end users throughout the development process [27].

### Objectives

We previously provided a proof of concept for a combinatorial approach of dietary recording that accounts for a wide range of dish variations [28,29]. The concept was shown to have potential for use by seniors [28], but design feedback was needed to improve the speed and accuracy of dietary reporting for this target group. Therefore, this research integrated a voice-input design enhancement for mobile app–based food intake reporting. The design approach integrated both voice and typical button interactions in handheld devices to develop two distinct prototypes for testing and comparison. This study investigated the practical usage experience of both apps and assessed their relative effectiveness for use by older adults.

## Methods

### General Overview of the Approach

We developed our voice-added design based on a user-centered design model [30] through research, ideation, and implementation steps. A review of the relevant literature and commercial apps was conducted, and team members engaged in extensive brainstorming for broad design ideas. One idea raised in the ideation stage was to allow voice-based intake recording. Our previously reported effort involved one-time voice reporting of food ingredients, portion size, cooking method, and other food attributes of a single dish. The initial prototype was reviewed by two senior dietitians, and testing results showed that one of the major obstacles was misrecognition of speech inputs. To reduce system complexity, we applied a design heuristics approach for simplicity [31,32] to propose two alternatives. The first alternative, voice-only reporting (VOR), decomposes the food contents of a dish into two parts. The user would then use voice inputs to describe these major content items, with additional items added later using traditional touch screen or voice input. The second alternative, voice-button reporting (VBR), adds a voice input feature to the existing touch-screen input procedure, based on our previous combinatorial food reporting concept. Major food ingredients were reported by voice input, and the remaining ingredients were reported using traditional touch screen–based user interaction.

### App Implementation

The two apps were implemented in the Android operating system for use on 7-inch tablet computers. The VOR app allows users to simultaneously verbally report food names and food attributes, whereas the VBR app allows users to verbally report food names and then select food attributes by clicking the optional buttons. The Google speech cloud service (Google, Inc) was used for continuous speech recognition in both apps. The developed interfaces included senior-friendly design elements, such as bigger buttons and text, a simple layout, and high-contrast colors. Based on recommended design guidelines for seniors [17], the two apps shared a common interface design, including placement of buttons, text, and icons. Clear and intuitive visual cues were used to facilitate user interaction.

### App Operation Overview

#### Voice-Only Reporting for a Dish With a Single Ingredient

**Figure 1:**
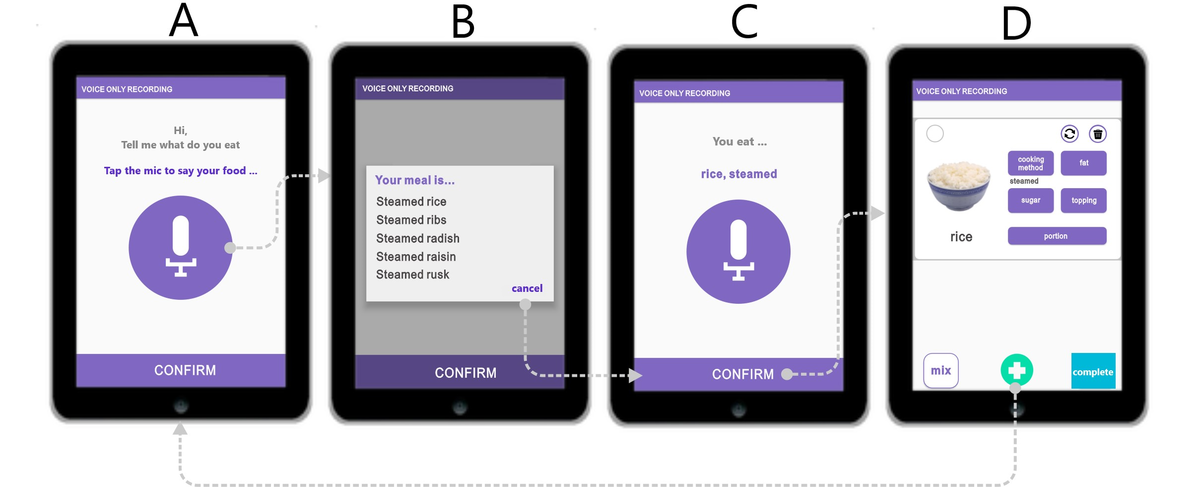
Voice-only reporting operation of a dish with a single ingredient, using steamed rice as an example.

Figure 1A shows the initial screen with the large “record” button used to activate speech recognition (Multimedia Appendix 1). The Chinese spoken language features many homophones, and recognized speech can be interpreted multiple ways. Figure 1B shows a menu of options resulting from the recognized speech, listed in descending order of confidence. The user selects the correct item from the list, and then, the system confirms the user’s selection (Figure 1C). Thereafter, the confirmed selection is displayed (Figure 1D).

As shown in Figure 1B, the user selects the desired response from the listed responses or selects “cancel” to rerecord the input. A lexical filter prunes the list of potential responses to only eliminate less relevant responses, thus avoiding presenting the user with a long list of unlikely possibilities. A grammatical function called “Food Grammar” was developed to parse the selected results by selecting predefined keywords from the app’s food name database based on four food attributes (method of cooking, sugar, fat, and topping). Words not kept by the filter are assumed to be food ingredients. For example, for “steamed rice,” “steamed” is identified as the method of cooking, while “rice” is recognized as an ingredient. Following the parsing operation, the app displays the recognized food contents as “rice, steamed” (Figure 1C). When the user clicks “confirm” (Figure 1C), the screen presents a food editing page with the name of the food, along with corresponding images and food attributes (Figure 1D). Clicking the “add more (+)” button located at the bottom center (Figure 1D) allows the user to input additional ingredients or dishes by returning to the initial step (Figure 1A). As shown in Figure 1D, there is an attribute adjustment feature to account for variations in the method of cooking (eg, salad, boiled, stewed, stir fried, fried, and deep fried), sugar content (eg, 0%, 25%, 50%, 75%, and 100%), types of milk, topping (eg, tapioca bubbles, coconut milk, and ice cream), and portion size (eg, plate, bowl, cup, and other).

#### Voice-Only Reporting for a Dish With Two or More Food Ingredients

**Figure 2:**
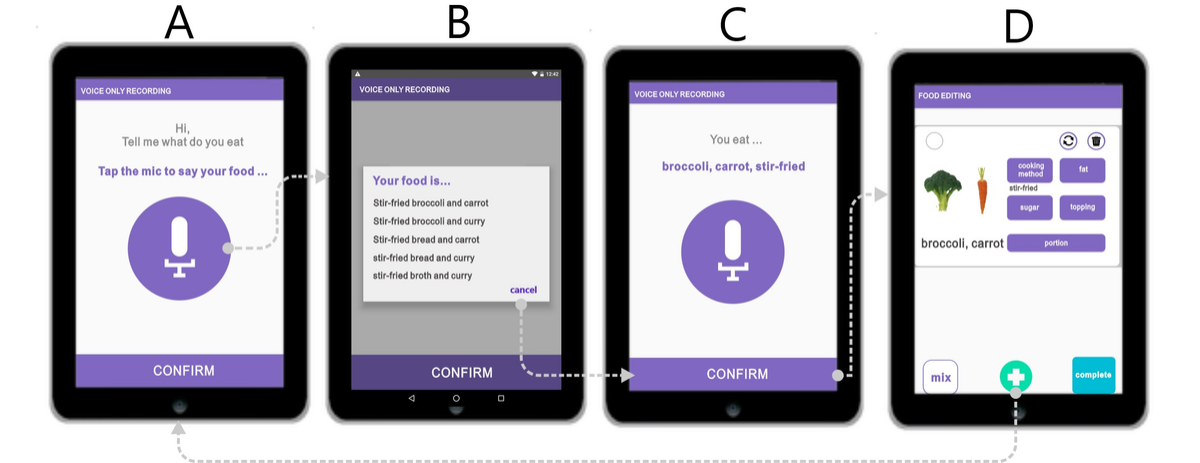
Voice-only reporting operation of a dish with two or three ingredients, using stir-fried broccoli with carrot as an example.

For dishes with two ingredients, the user verbally inputs the first ingredient followed by its associated food attribute, and then repeats the process for the second ingredient (Multimedia Appendix 2). As shown in Figure 2A, the user inputs the dish as follows: “broccoli, stir-fried, carrot.” In this case, the displayed list presents five possible alternatives (Figure 2B). The user selects the intended input from the list. After parsing, the selected result is shown (ie, broccoli, carrot, stir-fried; Figure 2C). The user then clicks “confirm” to move to the food editing page (Figure 2D). This input process is extended for dishes with two or more ingredients.

#### Voice-Button Reporting for a Dish With a Single Ingredient

**Figure 3:**
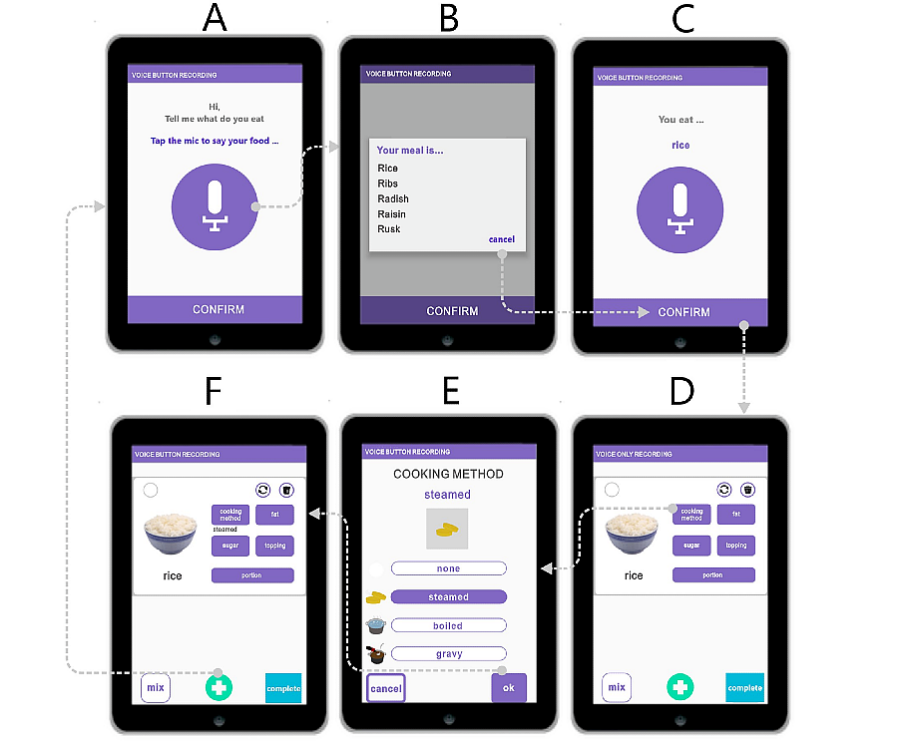
Voice-button reporting operation of a dish with a single ingredient, using steamed rice as an example.

Using VBR for a dish with a single ingredient, users first verbally input the name of the dish (Multimedia Appendix 3), for example, “rice” (Figure 3A). The food attributes are added in the following editing page by clicking one of the five buttons on the upper right (Figure 3D). The user thus adjusts the cooking method of “rice” by selecting “cooking method” (Figure 3D) and then selects the appropriate cooking method (eg, steamed; Figure 3E). The user clicks “OK” to then be presented with cooking method information (eg, steamed).

#### Voice-Button Reporting for a Dish With Two or More Ingredients

**Figure 4:**
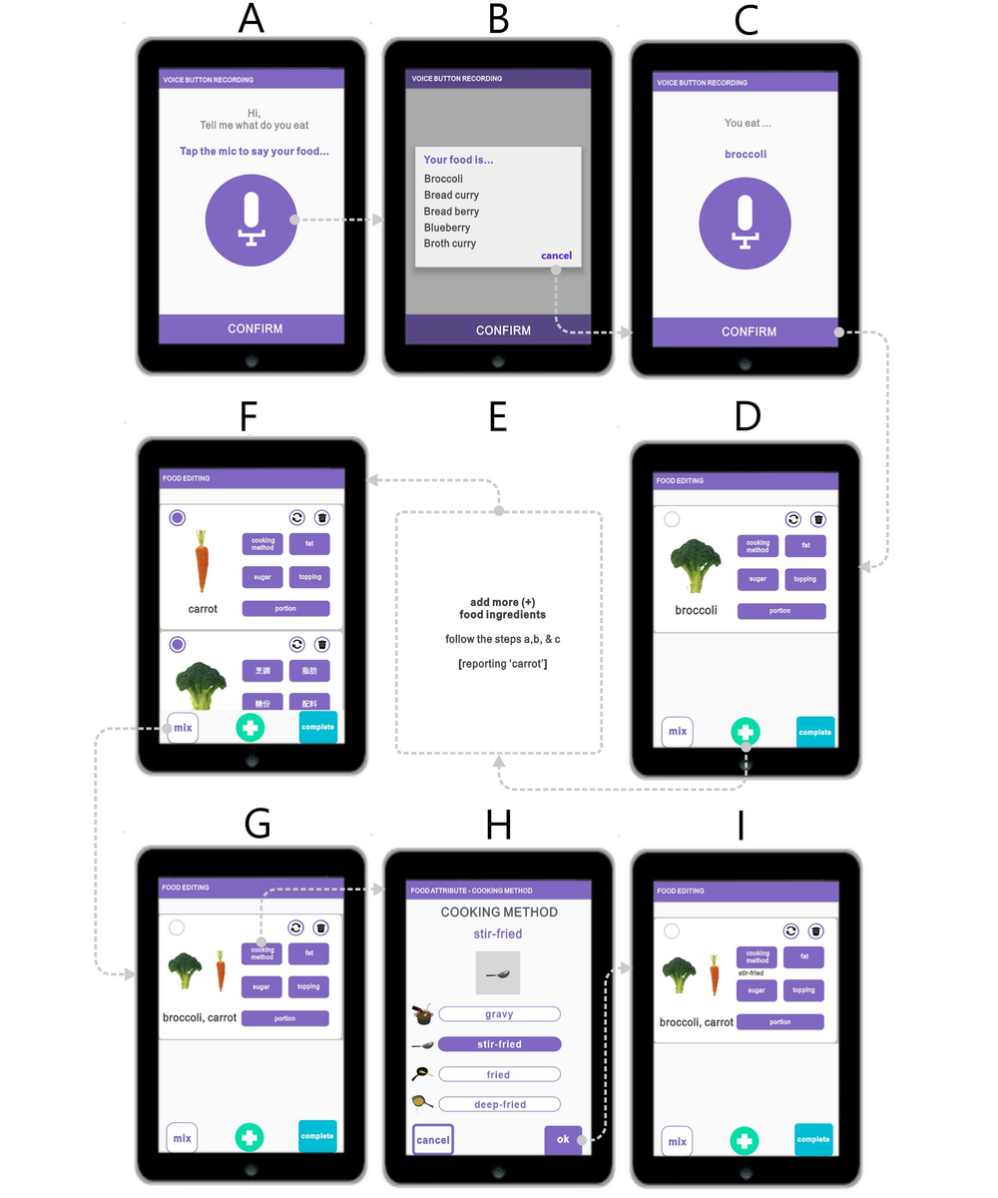
Voice-button reporting operation for a dish with two or more ingredients, using stir-fried broccoli with carrot as an example.

For dishes with two or three ingredients, the user begins by verbally inputting the first food ingredient and follows the first four steps (Figure 4A-D; Multimedia Appendix 4). The user then clicks the “add more (+)” button and continues to report all subsequent food ingredients. When all ingredients have been reported, the user clicks the “mix” button to assemble the dish. As shown in Figure 4, the user reports “broccoli” (Figure 4D) and “carrot” (Figure 4E). The user selects the desired food items (eg, broccoli and carrot) and clicks the “mix” button (Figure 4F) to create mixed-food information (Figure 4G). The user then chooses the desired cooking method and clicks “confirm” (Figure 4H). The “add more (+)” button also allows the user to report other dishes while the “complete” button finishes the food reporting session (Figure 4I).

### Study Design and Participant Recruitment

A parallel two-group randomized trial was designed to evaluate the relative effectiveness of the two apps in terms of reporting accuracy, task time, and user acceptance. The study protocol was reviewed by the Ethics Committee of Chang Gung Memorial Hospital and received Institutional Review Board approval (201900324B0). Recruitment was conducted through notices placed on designated bulletin boards in the Chang Gung Health and Culture Village retirement community located in northern Taiwan. Registration, schedule arrangement, and collection of background information were conducted through an online form. Biographic data were used to allocate participants into the VOR or VBR group. Self-reported baseline information included gender, age, BMI, experience in nutrition education, use of nutrition-related apps, cooking experience, and experience using mobile phones/tablets. Eligible participants were (1) aged from 60 to 90 years and (2) capable of reading and operating the app on their mobile phone. Participants currently under any form of dietary control, currently engaged in deliberate weight loss, or following a vegetarian diet were excluded. The assessment was conducted in a public area inside the community.

Dishes and beverages for the experiment were selected under the supervision of a senior nutritionist. The dishes were typical local Asian and Western-style foods. Three set meals involving 17 food items were used to represent breakfast, lunch, and dinner. Each set meal contained five food items (ie, a staple food, a main course, a dish with two ingredients, a dish with three ingredients, and a beverage). These set meals were presented on life-size colored food-photo boards (30 cm × 42 cm; photographed from above). Following previous research [28], to avoid disturbing variables, each dish was labeled (72 pt) above or below the food item (Multimedia Appendix 5).

### Sample Size Estimation

The sample size was based on our previous experience of customized dietary recording [28]. The mean difference for time required to complete the task using the two approaches was 4 seconds, with a SD of 5 seconds. Therefore, given a statistical power of 80% and a two-tailed α level of 5%, the minimum sample size required was 26 subjects each for VOR and VBR. Therefore, the minimum recruitment size was determined to be 52 subjects.

### Randomization

**Figure 5:**
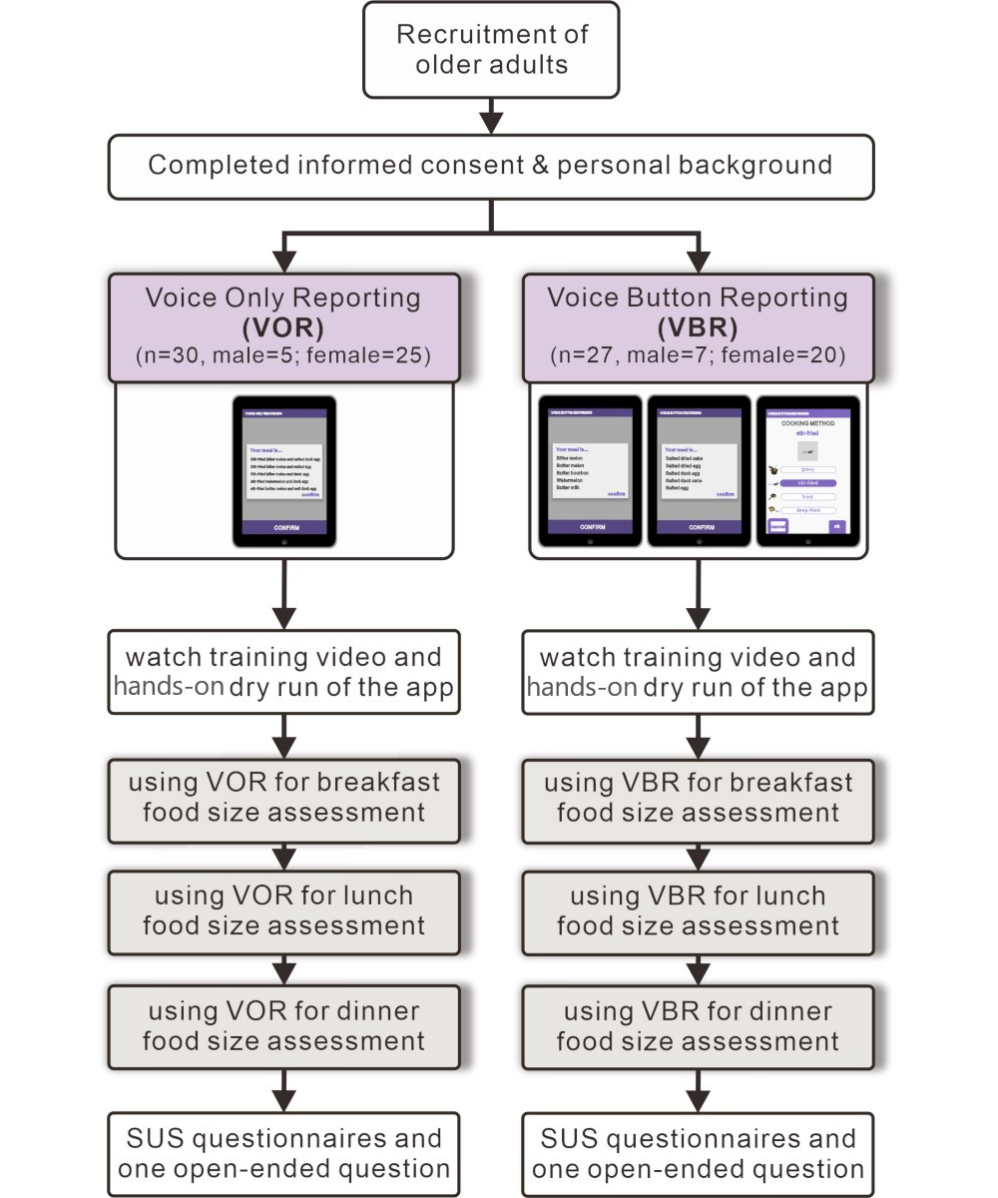
App evaluation flow using a randomized design. SUS: system usability scale.

A total of 57 senior participants were recruited and completed informed consent. SAS [33] was used to generate randomized lists of equal size with a 1:1 ratio for the two study arms, with 30 and 27 participants assigned to the VOR and VBR groups, respectively (Figure 5). The experiment was conducted with individual participants from each group in accordance with the randomization list.

### Evaluation Outcomes

The following three outcome types were assessed to evaluate the respective effectiveness of the two mobile apps for food reporting: accuracy, user operation time, and perception of efficacy.

#### Accuracy

An error was defined as the participant engaging in operating steps outside of those required to obtain the predefined answer. Possible error types of dish reporting were identified, and they have been described in the subsection “Error Types.” The rate of a specific error type was expressed as the error count divided by the total count. The error count was defined as the sum of participants with incorrect responses in the error type. The total count was calculated as the number of participants multiplied by the number of all dishes. In a specific error type, each participant’s reporting task might encounter more than one incorrect response, but was counted as one. The accuracy for error type was defined as the difference between the total count and error count divided by the total count.

#### Error Types

The error types were derived thematically for data analysis [34], which has been described in the subsection “Thematic Analysis.” Data were derived from each participant-system interaction, with the app automatically logging the participant’s selection of any functional buttons (eg, voice or mix button), and each interaction was tagged with a time stamp. The app also recorded the suggested results from the speech input along with the item subsequently selected and confirmed by the participant.

#### Task Duration

For VOR and VBR, the operating duration covered the time from when the participant began to input a food item until the participant tapped the “complete” button on the screen. For the VOR group, the task duration was calculated from the time the participant clicked the “voice” button to begin speaking to the time the participant clicked the “complete” button (Figure 1D and Figure 2D). For the VBR group, the task duration was calculated from the time the participant clicked the “voice” button to begin speaking to the time the participant completely mixed the multiple resulting ingredients and adjusted the cooking method (Figure 3F and Figure 4I). The mobile app automatically recorded the assessment duration of each participant.

### Perception

The system usability scale (SUS) [35] was used to measure participant perception, with a questionnaire of 10 items. Each item used a 5-point Likert scale from 1 (strongly disagree) to 5 (strongly agree). Following the study by Bangor et al [36], the mean SUS score with an adjective rating scale was used. Mean scores of 35.7, 50.9, 71.4, and 85.5 were rated as “poor,” “ok,” “good,” and “excellent,” respectively, in the adjective scales.

### Assessment Procedures

The experiment was carried out by two research assistants. Informed consent was explained to and obtained from each participant. All participants utilized the same hardware (ie, a 7-inch Android tablet). All participant trials were conducted on a single day. Each participant session was scheduled by appointment and implemented individually. Each participant was first trained by watching an instructional video demonstrating how the food reporting app could be used. The researchers then spent several minutes teaching each participant how to navigate the interface, to ensure familiarity with app operation and features. The experiment was arranged on the basis of a set meal, with each set meal involving a staple food, a main course, a dish with two ingredients, a dish with three ingredients, and a beverage. Having understood the meal concept, each participant conducted a “dry run,” which involved voice reporting of five food items (porridge, sausage, chicken egg, gluten with peanuts, and soy milk) on a photo board (Multimedia Appendix 5). A research assistant guided each participant to clearly pronounce the food name before conducting the food reporting. All participants were encouraged to become familiar with the app until they were confident with the app’s operation flow. Participants were informed that their time to task completion was also a performance to be considered.

Respondents were asked to report three set meals (breakfast, lunch, and dinner). The first set meal, representing breakfast, featured boiled rice porridge, grilled pork sausage, stir-fried chicken egg, wheat gluten stewed with peanuts, and soy milk. The second set meal, representing lunch, featured steamed rice, deep-fried chicken, stir-fried broccoli with carrots, stir-fried tofu with green beans, stir-fried cabbage with bacon and black mushrooms, and green tea. The third set meal, representing dinner, featured fried noodles, pan-fried mackerel, stir-fried bitter melon with bell peppers and carrots, and tea with milk. The meal tests were performed in sequence, with a rest of 1 to 3 minutes between each test. The total test time for each participant took about 1 hour, beginning from when the participant first clicked the voice record button, according to the procedure shown in the “General Overview of the Approach” section. All participants completed the assessment.

### Statistical Analysis

The chi-square test and *t* test were applied to examine the baseline characteristics of participants for categorical and continuous variables, respectively. According to our study endpoints, the accuracy between different groups was reported by the error proportion calculated as the number of errors by the total answer items. Second, the time duration for operating assessment was also employed for efficiency evaluation. As the time duration of reporting is a continuous variable, the *t* test was used to assess and compare the difference between the VOR and VBR groups. This comparison was also applied for dishes with different ingredients. SAS version 9.1.4 software (SAS Institute) was used to conduct all statistical analyses. All two-tailed statistical test results with a *P* value below .05 were considered to be statistically significant.

### Thematic Analysis

Following the study by Bree and Galagher [37], all analyses were conducted using a Microsoft Excel worksheet (Microsoft Inc). The data were analyzed by two research assistants. The themes (ie, the error types) were first identified. The process of identifying a possible theme included the following steps. First, each assistant investigated the data independently, highlighting and labeling mismatched operating tasks for each dish. Similar labels were clustered into a single error type with a common tag, such as “missing food names” and “missing cooking methods” (Multimedia Appendix 6). Similar error types could be further grouped into a theme, such as “trouble after reporting” and “trouble in selecting one among the choices.” When new error types or themes emerged, the overall network was revised accordingly. Discrepancies between the two assistants were discussed, and a consensus was reached under the supervision of the project leader.

## Results

### Participant Characteristics

A total of 68 participants were registered. Of these, 57 participants were scheduled and completed the experiment (Figure 5) and 11 failed to schedule an appointment. As shown in Table 1, 30 and 27 respondents were included in the VOR and VBR groups, respectively. The valid respondent pool had 21% (12/57) male and 79% (45/57) female participants, with an overall mean age of 73.92 (SD 1.48) years, where 12% (7/57) were aged 60 to 64 years, 40% (23/57) were aged 65 to 74 years, and the remaining 48% (27/57) were aged above 75 years. The mean BMI of all participants was 22.55 kg/m^2^ (SD 2.25). Nearly half (28/57, 49%) of the participants had a bachelor’s degree, followed by junior high school (12/57, 21%), senior high/vocational school (11/57, 19%), master’s degree (5/57, 9%), and others (1/57, 2%). Looking at previous relevant experience, 95% (54/57) of respondents reported having experience in cooking, while 68% (39/57) reported previous experience using mobile phones or tablets, 32% (18/57) had taken nutrition-related courses, and 7% (4/57) had used nutrition-related apps. Among the two groups, the baseline information did not reveal relevant differences, confirming randomized allocation.

**Table 1:** Participant characteristics in the voice-only reporting and voice-button reporting groups.

Variables		Total (N=57), n (%) or mean (SD)		Voice-only reporting group (n=30), n (%)		Voice-button reporting group (n=27), n (%)		*P* value	
**Gender**								.39	
	Male		12 (21%)		5 (17%)		7 (26%)		
	Female		45 (79%)		25 (83%)		20 (74%)		
**Age (years)^a^**								.15	
	≤64		7 (12%)		6 (20%)		1 (4%)		
	65-74		23 (40%)		10 (33%)		13 (48%)		
	≥75		27 (48%)		14 (47%)		13 (48%)		
BMI (kg/m^2^)^a^		22.55 (2.25)		22.63 (2.37)		22.45 (2.15)		.77	
**Education**								>.99	
	Junior high school		12 (21%)		6 (20%)		6 (22%)		
	Senior high/vocational school		11 (19%)		6 (20%)		5 (19%)		
	Bachelor’s degree		28 (49%)		14 (47%)		14 (52%)		
	Master’s degree		5 (9%)		3 (10%)		2 (7%)		
	Others		1 (2%)		1 (3%)		0 (0%)		
**Q1. Experience with** **nutrition-related courses**								.40	
	Yes		18 (32%)		8 (27%)		10 (37%)		
	No		39 (68%)		22 (73%)		17 (63%)		
**Q2. Experience with** **health education**								.58	
	Yes		17 (30%)		8 (27%)		9 (33%)		
	No		40 (70%)		22 (73%)		18 (67%)		
**Q3. Experience in cooking**								.60	
	Yes		54 (95%)		29 (97%)		25 (93%)		
	No		3 (5%)		1 (3%)		2 (7%)		
**Q4. Experience using** **nutrition-related apps**								>.99	
	Yes		4 (7%)		2 (7%)		2 (7%)		
	No		53 (93%)		28 (93%)		25 (93%)		
**Q5. Experience using** **mobile phones or tablets**								.38	
	Yes		39 (68%)		19 (63%)		20 (74%)		
	No		18 (32%)		11 (37%)		7 (26%)		

^a^Age and BMI data were analyzed with analysis of variance.

### Error Types and Overall Accuracy

Table 2 presents overall accuracy results in terms of correct and incorrect counts for the 17 food items. Eight error types were identified and categorized from the analysis of each participant’s food recording procedures (Multimedia Appendix 6). In the VOR group, error types were related to voice input (#1-3 and #5) and typical finger tap operations (#4 and #5-8). In the VBR group, error types were related to voice input (#1-3) and finger tap issues (#4-8). The error type “repeated pronunciations” (#5) was only relevant to VOR and “did not select the ‘mix’ button” (#7) only occurred in the VBR group. Among the eight error types, two error types (“missing cooking method(s)” [#4] and “did not select the ‘mix’ button” [#7]) showed significant differences (*P*<.001). The VOR group outperformed the VBR group in these two error types. In the VOR group, “no desirable choices” (#3) was the most commonly found error at a rate of 6.7% (28/420), followed by “incorrect selections in the list” (#6) at 3.8% (16/420) and “incorrect operation” (#8) at 2.6% (11/420). In the VBR group, “did not select the ‘mix’ button” (#7) was the most common error type at a rate of 10.3% (39/378), followed by “missing cooking method(s)” (#4) at 7.4% (28/378) and “no desirable choices” (#3) at 7.1% (27/378).

**Table 2:** Overall accuracy comparison of error types in the voice-only reporting and voice-button reporting groups.

Error type (correct/incorrect)^a^			Total (N=57), n (%)	Voice-only reporting group (n=30), n (%)	Voice-button reporting group (n=27), n (%)	*P* value
**(#1) Missing the first food names/syllable(s)^b^**						
	Correct		796 (99.7%)	418 (99.5%)	378 (100.0%)	.50
	Incorrect		2 (0.3%)	2 (0.5%)	0 (0.0%)	
**(#2) Missing the last food names/syllable(s)^c^**						
		Correct	792 (99.2%)	416 (99.0%)	376 (99.5%)	.69
		Incorrect	6 (0.8%)	4 (1.0%)	2 (0.5%)	
**(#3) No desirable choices^d^**						
		Correct	743 (93.1%)	392 (93.3%)	351 (92.9%)	.79
		Incorrect	55 (6.9%)	28 (6.7%)	27 (7.1%)	
**(#4) Missing cooking method(s)^e^**						
		Correct	766 (96.0%)	416 (99.0%)	350 (92.6%)	<.001
		Incorrect	32 (4.0%)	4 (1.0%)	28 (7.4%)	
**(#5) Repeated pronunciations^f^**						
		Correct	794 (99.5%)	416 (99.0%)	378 (100.0%)	.13
		Incorrect	4 (0.5%)	4 (1.0%)	0 (0.0%)	
**(#6) Incorrect selections in the list^g^**						
		Correct	771 (96.6%)	404 (96.2%)	367 (97.1%)	.48
		Incorrect	27 (3.4%)	16 (3.8%)	11 (2.9%)	
**(#7) Did not select ‘mix’ button^h^**						
		Correct	759 (95.1%)	420 (100.0%)	339 (89.7%)	<.001
		Incorrect	39 (4.9%)	0 (0.0%)	39 (10.3%)	
**(#8) Incorrect operation^i^**						
		Correct	775 (97.1%)	409 (97.4%)	366 (96.8%)	.64
		Incorrect	23 (2.9%)	11 (2.6%)	12 (3.2%)	

^a^Three items in beverage were not counted as no error types were found. Fourteen out of the 17 food items were included.

^b^#1 Missing first food name/syllable(s): After verbal reporting, the presented answer list did not include the first food name or the first syllable(s) of the food names.

^c^#2 Missing last food name/syllable(s): After verbal reporting, the presented answer list did not include the last food name or the last syllable(s) of the food names after voice reporting.

^d^#3 No desirable choices: After verbal reporting, the presented answer list did not present the desired food name or cooking method.

^e^#4 Missing cooking method(s): After verbal reporting, the presented answer list did not include the desired cooking method(s).

^f^#5 Repeated pronunciations: The presented answer list showed repeated pronunciations of food names and/or food attributes after voice reporting.

^g^#6 Incorrect selections in the list: Participant had trouble accurately tapping the desired choice (click interaction), leading to incorrect selection in the answer list.

^h^#7 Did not select the ‘mix’ button: Trouble before dish completion (click interaction). The user did not tap the “mix” button to complete dishes with two or three ingredients.

^i^#8 Incorrect operations: Incorrect operation procedure.

### Accuracy and Trial and Error for Each Dish

The results are presented in terms of dish complexity (ie, number of ingredients) (Table 3). In addition, no errors were found for the three beverage items in either test group.

**Table 3:** Accuracy comparison of each food item in the voice-only reporting and voice-button reporting groups.

Food item and error type			Total (N=57), n (%)	Voice-only reporting group (n=30), n (%)	Voice-button reporting group (n=27), n (%)
**Staple food**					
	**Boiled rice porridge**				
		#1^a^	0 (0%)	0 (0%)	0 (0%)
		#2^b^	0 (0%)	0 (0%)	0 (0%)
		#3^c^	1 (2%)	0 (0%)	1 (4%)
		#4^d^	3 (5%)	1 (3%)	2 (7%)
		#5^e^	0 (0%)	0 (0%)	0 (0%)
		#6^f^	0 (0%)	0 (0%)	0 (0%)
		#7^g^	0 (0%)	0 (0%)	0 (0%)
		#8^h^	0 (0%)	0 (0%)	0 (0%)
	**Steamed white rice**				
		#1	0 (0%)	0 (0%)	0 (0%)
		#2	0 (0%)	0 (0%)	0 (0%)
		#3	3 (5%)	2 (7%)	1 (4%)
		#4	6 (11%)	0 (0%)	6 (22%)
		#5	1 (2%)	1 (3%)	0 (0%)
		#6	0 (0%)	0 (0%)	0 (0%)
		#7	0 (0%)	0 (0%)	0 (0%)
		#8	0 (0%)	0 (0%)	0 (0%)
	**Stir-fried noodle**				
		#1	0 (0%)	0 (0%)	0 (0%)
		#2	0 (0%)	0 (0%)	0 (0%)
		#3	0 (0%)	0 (0%)	0 (0%)
		#4	2 (4%)	0 (0%)	2 (7%)
		#5	0 (0%)	0 (0%)	0 (0%)
		#6	0 (0%)	0 (0%)	0 (0%)
		#7	0 (0%)	0 (0%)	0 (0%)
		#8	1 (2%)	0 (0%)	1 (4%)
**Main course**					
	**Grilled pork sausage**				
		#1	0 (0%)	0 (0%)	0 (0%)
		#2	0 (0%)	0 (0%)	0 (0%)
		#3	2 (4%)	1 (3%)	1 (4%)
		#4	3 (5%)	1 (3%)	2 (7%)
		#5	0 (0%)	0 (0%)	0 (0%)
		#6	2 (4%)	1 (3%)	1 (4%)
		#7	0 (0%)	0 (0%)	0 (0%)
		#8	2 (4%)	1 (3%)	1 (4%)
	**Stir-fried chicken egg**				
		#1	0 (0%)	0 (0%)	0 (0%)
		#2	0 (0%)	0 (0%)	0 (0%)
		#3	0 (0%)	0 (0%)	0 (0%)
		#4	2 (4%)	0 (0%)	2 (7%)
		#5	0 (0%)	0 (0%)	0 (0%)
		#6	3 (5%)	2 (7%)	1 (4%)
		#7	0 (0%)	0 (0%)	0 (0%)
		#8	1 (2%)	0 (0%)	1 (4%)
	**Deep-fried chicken leg**				
		#1	0 (0%)	0 (0%)	0 (0%)
		#2	0 (0%)	0 (0%)	0 (0%)
		#3	2 (4%)	0 (0%)	2 (7%)
		#4	2 (4%)	0 (0%)	2 (7%)
		#5	0 (0%)	0 (0%)	0 (0%)
		#6	3 (5%)	0 (0%)	3 (11%)
		#7	0 (0%)	0 (0%)	0 (0%)
		#8	1 (2%)	0 (0%)	1 (4%)
	**Pan-fried mackerel**				
		#1	0 (0%)	0 (0%)	0 (0%)
		#2	0 (0%)	0 (0%)	0 (0%)
		#3	10 (18%)	6 (20%)	4 (15%)
		#4	2 (4%)	0 (0%)	2 (7%)
		#5	0 (0%)	0 (0%)	0 (0%)
		#6	1 (2%)	1 (3%)	0 (0%)
		#7	0 (0%)	0 (0%)	0 (0%)
		#8	1 (2%)	1 (3%)	0 (0%)
**Dishes with two ingredients**					
	**Stewed wheat gluten with peanuts**				
		#1	1 (2%)	1 (3%)	0 (0%)
		#2	1 (2%)	1 (3%)	0 (0%)
		#3	19 (33%)	12 (40%)	7 (26%)
		#4	3 (5%)	0 (0%)	3 (11%)
		#5	2 (4%)	2 (7%)	0 (0%)
		#6	12 (21%)	10 (33%)	2 (7%)
		#7	14 (25%)	0 (0%)	14 (52%)
		#8	6 (11%)	2 (7%)	4 (15%)
	**Stir-fried broccoli with carrot**				
		#1	0 (0%)	0 (0%)	0 (0%)
		#2	0 (0%)	0 (0%)	0 (0%)
		#3	1 (2%)	0 (0%)	1 (4%)
		#4	1 (2%)	0 (0%)	1 (4%)
		#5	0 (0%)	0 (0%)	0 (0%)
		#6	2 (4%)	0 (0%)	2 (7%)
		#7	3 (5%)	0 (0%)	3 (11%)
		#8	0 (0%)	0 (0%)	0 (0%)
	**Stir-fried tofu with green bean**				
		#1	0 (0%)	0 (0%)	0 (0%)
		#2	1 (2%)	1 (3%)	0 (0%)
		#3	2 (4%)	1 (3%)	1 (4%)
		#4	1 (2%)	0 (0%)	1 (4%)
		#5	0 (0%)	0 (0%)	0 (0%)
		#6	0 (0%)	0 (0%)	0 (0%)
		#7	7 (12%)	0 (0%)	7 (26%)
		#8	1 (2%)	1 (3%)	0 (0%)
	**Stir-fried chicken egg with tomato**				
		#1	1 (2%)	1 (3%)	0 (0%)
		#2	0 (0%)	0 (0%)	0 (0%)
		#3	0 (0%)	0 (0%)	0 (0%)
		#4	1 (2%)	0 (0%)	1 (4%)
		#5	0 (0%)	0 (0%)	0 (0%)
		#6	1 (2%)	0 (0%)	1 (4%)
		#7	5 (9%)	0 (0%)	5 (19%)
		#8	2 (4%)	0 (0%)	2 (7%)
	**Stir-fried bitter melon with salted duck egg**				
		#1	0 (0%)	0 (0%)	0 (0%)
		#2	1 (2%)	0 (0%)	1 (4%)
		#3	7 (12%)	2 (7%)	5 (19%)
		#4	1 (2%)	0 (0%)	1 (4%)
		#5	0 (0%)	0 (0%)	0 (0%)
		#6	0 (0%)	0 (0%)	0 (0%)
		#7	3 (5%)	0 (0%)	3 (11%)
		#8	0 (0%)	0 (0%)	0 (0%)
**Dishes with three ingredients**					
	**Stir-fried cabbage with bacon and black fungus**				
		#1	0 (0%)	0 (0%)	0 (0%)
		#2	1 (2%)	0 (0%)	1 (4%)
		#3	5 (9%)	2 (7%)	3 (11%)
		#4	4 (7%)	2 (7%)	2 (7%)
		#5	0 (0%)	0 (0%)	0 (0%)
		#6	2 (4%)	2 (7%)	0 (0%)
		#7	5 (9%)	0 (0%)	5 (19%)
		#8	5 (9%)	4 (13%)	1 (4%)
	**Stir-fried dry bean curd with bell pepper and carrot**				
		#1	0 (0%)	0 (0%)	0 (0%)
		#2	2 (4%)	2 (7%)	0 (0%)
		#3	3 (5%)	2 (7%)	1 (4%)
		#4	1 (2%)	0 (0%)	1 (4%)
		#5	1 (2%)	1 (3%)	0 (0%)
		#6	1 (2%)	0 (0%)	1 (4%)
		#7	2 (4%)	0 (0%)	2 (7%)
		#8	3 (5%)	2 (7%)	1 (4%)
**Beverage**					
	**Soymilk**				
		#1	0 (0%)	0 (0%)	0 (0%)
		#2	0 (0%)	0 (0%)	0 (0%)
		#3	0 (0%)	0 (0%)	0 (0%)
		#4	0 (0%)	0 (0%)	0 (0%)
		#5	0 (0%)	0 (0%)	0 (0%)
	**Green tea**				
		#1	0 (0%)	0 (0%)	0 (0%)
		#2	0 (0%)	0 (0%)	0 (0%)
		#3	0 (0%)	0 (0%)	0 (0%)
		#4	0 (0%)	0 (0%)	0 (0%)
		#5	0 (0%)	0 (0%)	0 (0%)
	**Milk tea**				
		#1	0 (0%)	0 (0%)	0 (0%)
		#2	0 (0%)	0 (0%)	0 (0%)
		#3	0 (0%)	0 (0%)	0 (0%)
		#4	0 (0%)	0 (0%)	0 (0%)
		#5	0 (0%)	0 (0%)	0 (0%)

^a^#1 Missing first food name/syllable(s): After verbal reporting, the presented answer list did not include the first food name or the first syllable(s) of the food names.

^b^#2 Missing last food name/syllable(s): After verbal reporting, the presented answer list did not include the last food name or the last syllable(s) of the food names after voice reporting.

^c^#3 No desirable choices: After verbal reporting, the presented answer list did not present the desired food name or cooking method.

^d^#4 Missing cooking method(s): After verbal reporting, the presented answer list did not include the desired cooking method(s).

^e^#5 Repeated pronunciations: The presented answer list showed repeated pronunciations of food names and/or food attributes after voice reporting.

^f^#6 Incorrect selections in the list: Participant had trouble accurately tapping the desired choice (click interaction), leading to incorrect selection in the answer list.

^g^#7 Did not select the ‘mix’ button: Trouble before dish completion (click interaction). The user did not tap the “mix” button to complete dishes with two or three ingredients.

^h^#8 Incorrect operations: Incorrect operation procedure.

#### Dishes With a Single Ingredient

These food items included three staple foods and four main courses. The VOR group featured fewer “missing cooking method(s)” errors (n=2) than the VBR group (n=18). The two groups showed similar results for error types #3 and #6. Both groups showed elevated error rates for error type #3 for pan-fried mackerel (n=6 in the VOR group; n=4 in the VBR group). In the VBR group, the incidence of error type #4 was higher for steamed white rice (n=6), but low for boiled rice porridge (n=2), stir-fried noodle (n=2), grilled pork sausage (n=2), stir-fried chicken egg (n=2), and deep-fried chicken leg (n=2). The incidences of other error types were relatively low.

#### Dishes With Two Ingredients

Five dishes included two ingredients. In the VOR group, error type #3 was more frequent for stewed wheat gluten with peanuts (n=12, 40%) and error type #6 was more frequent for stewed wheat gluten with peanuts (n=10, 33%). In the VBR group, error type #7 was more frequent for stewed wheat gluten with peanuts (n=14, 52%), stir-fried tofu with green bean (n=7, 26%), and stir-fried chicken egg with tomato (n=5, 19%). In the VBR group, the frequency of error #3 was also relatively high for stir-fried bitter melon with salted duck egg (n=5, 19%). The incidences of other error types were relatively low in both groups.

#### Dishes With Three Ingredients

Three dishes were tested. In both the VOR and VBR groups, error type #3 occurred for two dishes, that is, stir-fried cabbage with bacon and black fungus (n=2 and n=3, respectively) and stir-fried dry bean curd with bell pepper and carrot (n=2 and n=1, respectively). Error type #7 had a higher incidence in the VBR group for stir-fried cabbage with bacon and black fungus (n=5), while error type #8 occurred frequently in the VOR group for stir-fried cabbage with bacon and black fungus (n=4). The incidences of other error types were relatively low in both groups.

### Time Efficiency

Table 4 shows the time participants needed to complete the reporting task for the 17 food items. The results showed that the VOR group significantly outperformed the VBR group in terms of time efficiency (*P*<.001), with statistically significant advantages for all food items, aside from beverages. The per task operation time in the VOR group ranged from 9 to 42 seconds, as opposed to 8 to 70 seconds (mean 37.52 s) in the VBR group.

**Table 4:** Reporting time in the voice-only reporting and voice-button reporting groups.

Food item		Reporting time (s)			*P* value
		Total (N=57), mean (SD)	Voice-only reporting group (n=30), mean (SD)	Voice-button reporting group (n=27), mean (SD)	
**Staple food**					
	Boiled rice porridge	20.44 (16.20)	10.50 (4.57)	31.49 (17.36)	<.001
	Steamed rice	14.37 (8.19)	10.11 (4.98)	19.11 (8.53)	<.001
	Stir-fried noodle	14.20 (10.75)	8.67 (3.45)	20.35 (12.69)	<.001
**Main course**					
	Grilled pork sausage	26.39 (38.58)	12.20 (6.70)	42.15 (51.63)	.006
	Deep-fried chicken egg	16.54 (10.47)	11.46 (8.82)	22.17 (9.32)	<.001
	Fried chicken leg	16.80 (13.53)	8.99 (2.97)	25.48 (15.36)	<.001
	Pan-fried mackerel	20.24 (12.69)	15.01 (11.23)	26.06 (11.81)	<.001
**Dishes with two ingredients**					
	Stewed wheat gluten with peanuts	51.73 (32.09)	42.38 (28.05)	62.11 (33.58)	.02
	Stir-fried broccoli with carrot	36.20 (36.30)	12.68 (4.17)	62.34 (38.34)	<.001
	Stir-fried tofu with green bean	30.98 (27.86)	10.80 (4.90)	53.41 (25.56)	<.001
	Stir-fried chicken egg with tomato	32.32 (28.73)	12.32 (6.32)	54.55 (27.54)	<.001
	Stir-fried bitter melon with salted duck egg	33.90 (32.36)	12.39 (5.11)	57.80 (33.16)	<.001
**Dishes with three ingredients**					
	Stir-fried cabbage with bacon and black fungus	44.39 (35.08)	21.23 (19.68)	70.13 (30.20)	<.001
	Stir-fried dry bean curd with bell pepper and carrot	41.81 (33.76)	16.62 (8.42)	69.80 (28.82)	<.001
**Beverage**					
	Soymilk	10.82 (7.20)	9.91 (6.31)	11.86 (8.11)	.31
	Green tea	8.76 (2.66)	8.86 (3.07)	8.66 (2.16)	.78
	Milk tea	9.64 (6.26)	10.81 (8.35)	8.35 (1.84)	.13

#### Time Efficiency for Dishes With One Ingredient

In the VOR group, the operation time ranged from 8 to 15 seconds per task, with pan-fried mackerel taking the longest time (mean 15.01, SD 11.23 s). In the VBR group, the operation time ranged from 19 to 41 seconds per task (mean 26.70 s), with grilled pork sausage taking the longest time (mean 42.15, SD 51.63 s). On average, the performance of the VOR group was roughly twice that of the VBR group.

#### Time Efficiency for Dishes With Two Ingredients

In the VOR group, four of the five dishes took 11 to 13 seconds, while stewed wheat gluten with peanuts took over 42 seconds. In the VBR group, the operation time ranged from 50 to 60 seconds, with stewed wheat gluten with peanuts taking over 60 seconds.

#### Time Efficiency for Dishes With Three Ingredients

The operation time in the VOR group ranged from 16 to 23 seconds, as opposed to 67 to 68 seconds in the VBR group.

#### Time Efficiency for Beverages

Both groups showed similar reporting operation time performance for beverages, with the VOR group taking 9 to 11 seconds per task, as opposed to 9 to 12 seconds in the VBR group.

### System Usability Scale and Subjective Perception

Table 5 summarizes the SUS score and its two divisions in terms of usability and learnability. Overall scores showed no significant differences between the VOR and VBR groups (*P*=.20), but both exceeded the mean score of 71.4, indicating that the participants in both groups considered the app as being “good” to “excellent.” In terms of learnability scores, the two groups showed a marginally significant difference (*P*=.06), suggesting that users found the VBR app slightly more difficult to learn to use.

**Table 5:** System usability scale and subjective perception in the voice-only reporting and voice-button reporting groups.

Score^a,b,c^	Voice-only reporting group (n=30), mean (SD)	Voice-button reporting group (n=27), mean (SD)	*P* value
Overall score	83.80 (9.49)	80.44 (10.25)	.20
Usability score	83.58 (9.57)	81.57 (9.69)	.43
Learnability score	84.67 (14.56)	75.93 (20.24)	.06

^a^Questionnaires were presented in Chinese.

^b^The mean score of the system usability scale with adjective ratings were as follows: 35.7 (“poor”), 50.9 (“ok”), 71.4 (“good”), and 85.5 (“excellent”).

^c^The questionnaire’s Cronbach α for voice-only reporting (α=.77) and voice-button reporting (α=.78) exceeded .70, indicating good internal consistency and reliability.

## Discussion

### Principal Findings

Two different voice-reporting designs were compared to investigate their respective effectiveness for food reporting among elderly users. VOR was designed to use verbal inputs for food names and attributes. VBR was operated through a sequential process of voice input and button tapping to report dietary intake. Experimental results showed the respective advantages and disadvantages of the two design concepts for authentic food reporting by older people. Our evidence-based findings provide insights into the relative usability of voice input in the food intake reporting context. The implications of these findings are discussed below, along with suggestions for further system improvement through the integration of voice input in the mobile health domain.

### Accuracy Analysis: VOR Versus VBR

The eight error types identified in this research provide a useful reference for potential types of errors that will be encountered in voice-enabled user dietary intake interactions. The better performance of VOR for error types #4 and #7 indicates that VOR has the potential to provide greater accuracy in food reporting. Participants experienced error type #3 in both the VOR and VBR groups. This error is related to phoneme and syllable-based speech recognition issues, and food names or food attributes with similar phonemes tend to have lower recognition accuracy. For instance, in the VOR and VBR groups, error type #3 was most prevalent for “stewed wheat gluten with peanuts,” and the Chinese term for “wheat gluten” (miàn cháng) was frequently misunderstood as “miàn chá.” Participants also experienced a higher incidence of recognition errors for cooking methods, for example, lǔ (stew) was misrecognized as rǔ (milk), zhǔ (boil), and fǔ (rotten), contributing to the system’s difficulty in accurately recognizing “stewed wheat gluten with peanuts.” In addition, incorrect recognition results were found for food names such as “peanut,” “bacon,” and “salted duck egg,” possibly because seniors have greater difficulty articulating nasal vowels [38]. One thing worth further investigating is the impact of the additional button tap required in VBR to identify food attributes. In contrast, VOR relied solely on verbal inputs and was designed to accommodate longer utterances, thus reducing the impact of error type #3.

The error type “did not select the ‘mix’ button” (#7) had the highest frequency among all errors in the VBR group and was specific to the item categories “dishes with two ingredients” (23.7%) and “dishes with three ingredients” (13.0%). This error may result from the app imposing cognitive overloading, as advanced age is associated with a decline in working memory [39]. VOR was designed to require fewer button taps, thus reducing opportunities for missing taps. To address this issue, future app designs should provide additional user training or design improvements [17] to prompt participants to remember to tap the required buttons.

The error “incorrect selections in the list” (#6) occurred with relatively high frequency in both groups (ranked second in the VOR group and third in the VBR group). This error is related to the user selecting the correct answer from a list of one to five possible choices, and could be explained by issues related to multimodal interaction in hand-eye coordination and speech input [40]. Our previous study [28] also addressed this issue for older adults, and system usability performance could be improved through improvements to the visual layout, increase in font size, or further improvements to user interaction design. Another multimodal interaction issue occurred in the error type “missing first food name/syllable(s)” (#1). However, this error occurred rarely. Among the 17 reporting task items, the three beverage items did not incur any errors, suggesting that their relative simplicity made them relatively easy to report accurately in both apps.

### Time Efficiency Analysis: VOR Versus VBR

For dishes with two ingredients, the need to tap buttons in the VBR group contributed to time efficiency up to five times worse than that in the VOR group (eg, 51.63 vs 10.67 s for “stir-friend tofu with green bean”). Aside from the beverage items, VOR consistently outperformed VBR in terms of time efficiency. The slower response time of VBR may be due to the need for additional button tapping to move between pages, and time spent on trial and error to obtain the correct food names or food attributes.

### Participant Perception

The overall SUS score exceeded the adjective rating of “good,” indicating that the participants considered the two apps to be useful. The high accuracy rate achieved by the two groups may conform with the high overall SUS scores. The significant time difference (*P*=.06) for task completion might reflect the marginally significant difference in the learnability component of the SUS model.

### Use of Voice-Added Interfaces for Seniors

Some previous studies [17,26] suggested that senior citizens find button tapping-based interfaces on smartphones to be challenging. Following a previous report [28], the design of the apps developed for this study sought to simplify interface interaction as much as possible, to better suit the needs of senior users. The VOR interface only required one step for dish reporting, while the VBR interface required three steps (report food name, add food attribute, and mix the dish). In the VBR group, participants experienced errors in clicking food attributes (eg, error type #4, “missing cooking method(s)”). Additionally, some participants in the VBR group tended to forget or neglected the add food attributes and mix dish steps, thus reducing dietary intake reporting accuracy. However, neither of these steps was required in the VOR group. The situation is similar to reporting multiple ingredients or methods of cooking in that it requires multiple word inputs, but it did not reduce VOR performance. The results showed that VOR and VBR had a similar frequency of error type #3 for dishes with multiple ingredients. Moreover, VOR had significantly better time efficiency than VBR (*P*<.001). Previous research [25] has suggested that voice-enabled interfaces could potentially reduce barriers to use by elderly people having vision and motor disabilities. The voice-enabled interface provided in VOR optimizes this approach by minimizing button tapping requirements. Although voice-enabled interfaces may offer improved accessibility for older users, some issues still need to be investigated. This study found that participants using the voice input encountered recognition errors, and certain dish reporting tasks took a relatively long time to complete.

### Limitations and Future Research

The experiments were conducted under laboratory conditions using a predetermined list of dishes and beverages. Participants were recruited from a retirement community; thus, further tests are required using different target populations (eg, seniors with specific chronic illnesses) whose results may differ from those of the groups tested here. The intended use case [41] in this research was to perform meal reporting with the use of the voice-added intake app, assuming users are familiar with the food ingredients and cooking methods of each dish. To better reflect realistic eating situations, future research should consider field user experience testing conducted in authentic settings. A wider range of authentic Asian and Western-style dishes and longer testing periods could also be included. Additional studies are needed to confirm the value of integrating voice inputs for food reporting. Further comparisons of the performance of voice-enabled and traditional interfaces under various eating contexts are needed. In addition, the idea of applying voice input to support existing dietary intake reporting apps could be explored to determine how and to what degree such integration improves usability. Further work also needs to include additional variables (eg, serving portion size, sugar and fat content, and toppings).

### Conclusion

Experimental results showed that, while users assessed both VOR and VBR as having similar utility, VOR had better accuracy and time efficiency, making it a better candidate for food reporting by seniors. The design of VOR is superior to that of VBR in that it relies solely on voice input for food intake reporting and does not require additional button taps. Experimental results showed that speech recognition results for certain food items have reduced recognition accuracy, and both groups evidenced challenges in selecting the desired items from the postvoice input suggestion menu. The user experience assessment results for the two apps developed for this research provide a useful empirical reference for the development of high usability consumer apps for dietary monitoring among elderly people. Further studies are required, including investigations involving authentic dining environments with real-world meal options, along with full-scale randomized controlled trials to assess test efficacy.

## Appendix

Multimedia Appendix 1 Mobile app voice-only reporting of a dish with one ingredient.

Multimedia Appendix 2 Mobile app voice-only reporting of a dish with two or more ingredients.

Multimedia Appendix 3 Mobile app voice-button reporting of a dish with one ingredient.

Multimedia Appendix 4 Mobile app voice-button reporting of a dish with two or more ingredients.

Multimedia Appendix 5 Images of three set meals.

Multimedia Appendix 6 Error types of dietary intake using a voice reporting approach.

Multimedia Appendix 7 CONSORT eHealth checklist.

## Abbreviations

SUS: system usability scale
VBR: voice-button reporting
VOR: voice-only reporting
