# Supplementary material for: Design and Usability Evaluation of Mobile Voice-Added Food Reporting for Elderly People: Randomized Controlled Trial
Source: JMIR Mhealth Uhealth. 2020 Sep 28;8(9):e20317. doi: 10.2196/20317 (PMC7551114; doi:10.2196/20317)
Supplement: Multimedia Appendix 5 [file mhealth_v8i9e20317_app5.pdf]

Pre-test Set

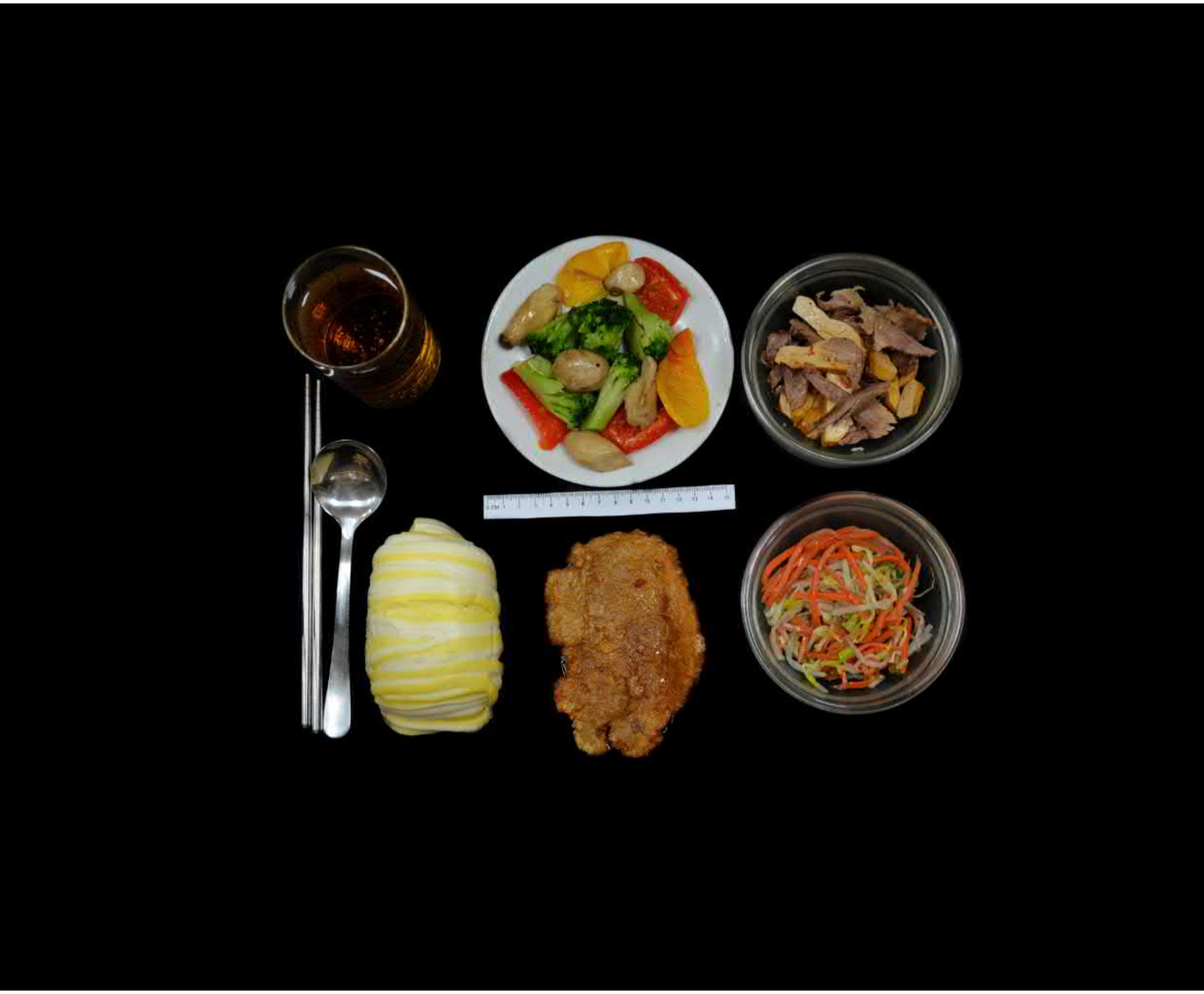

Breakfast Set

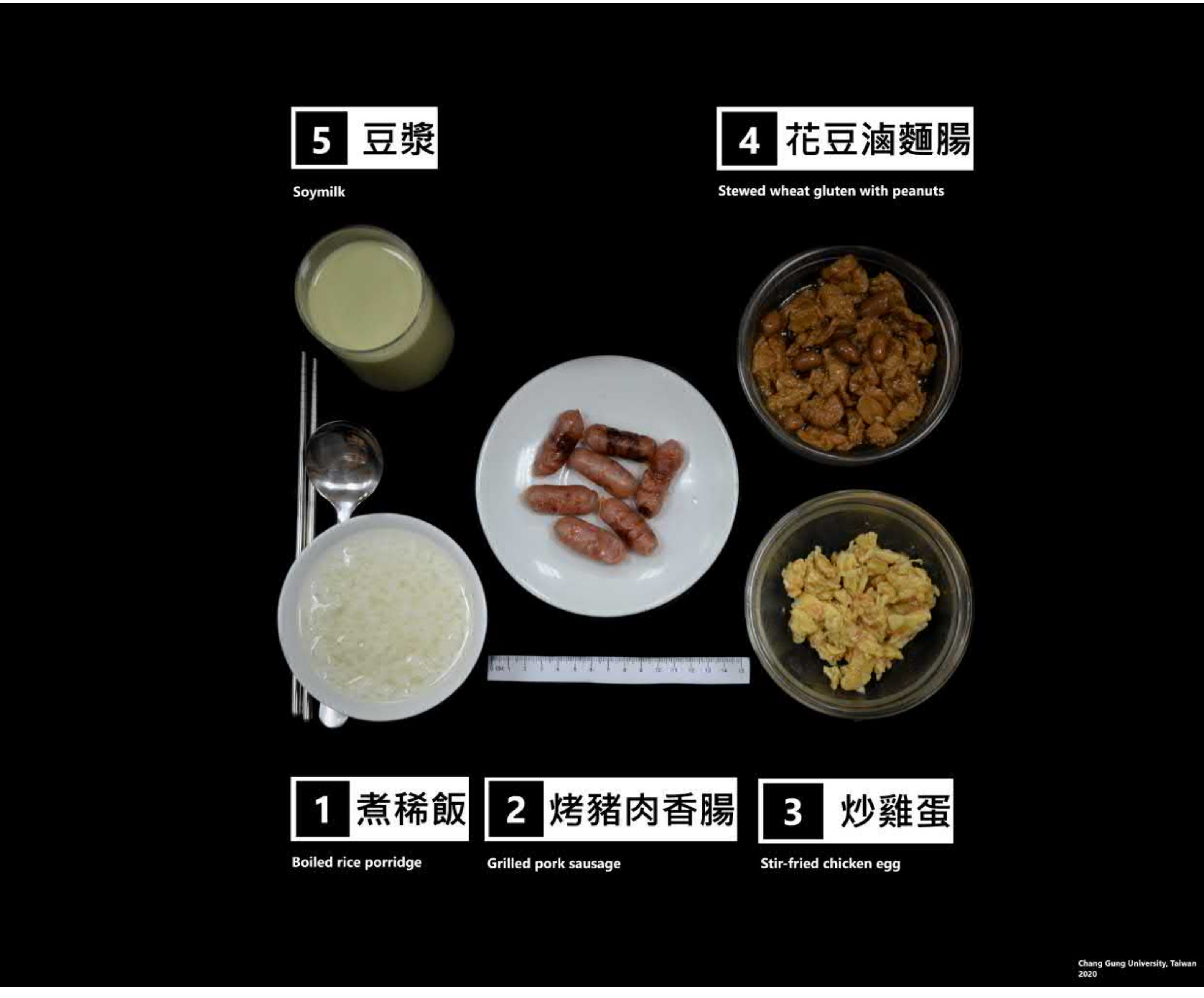

Lunch Set

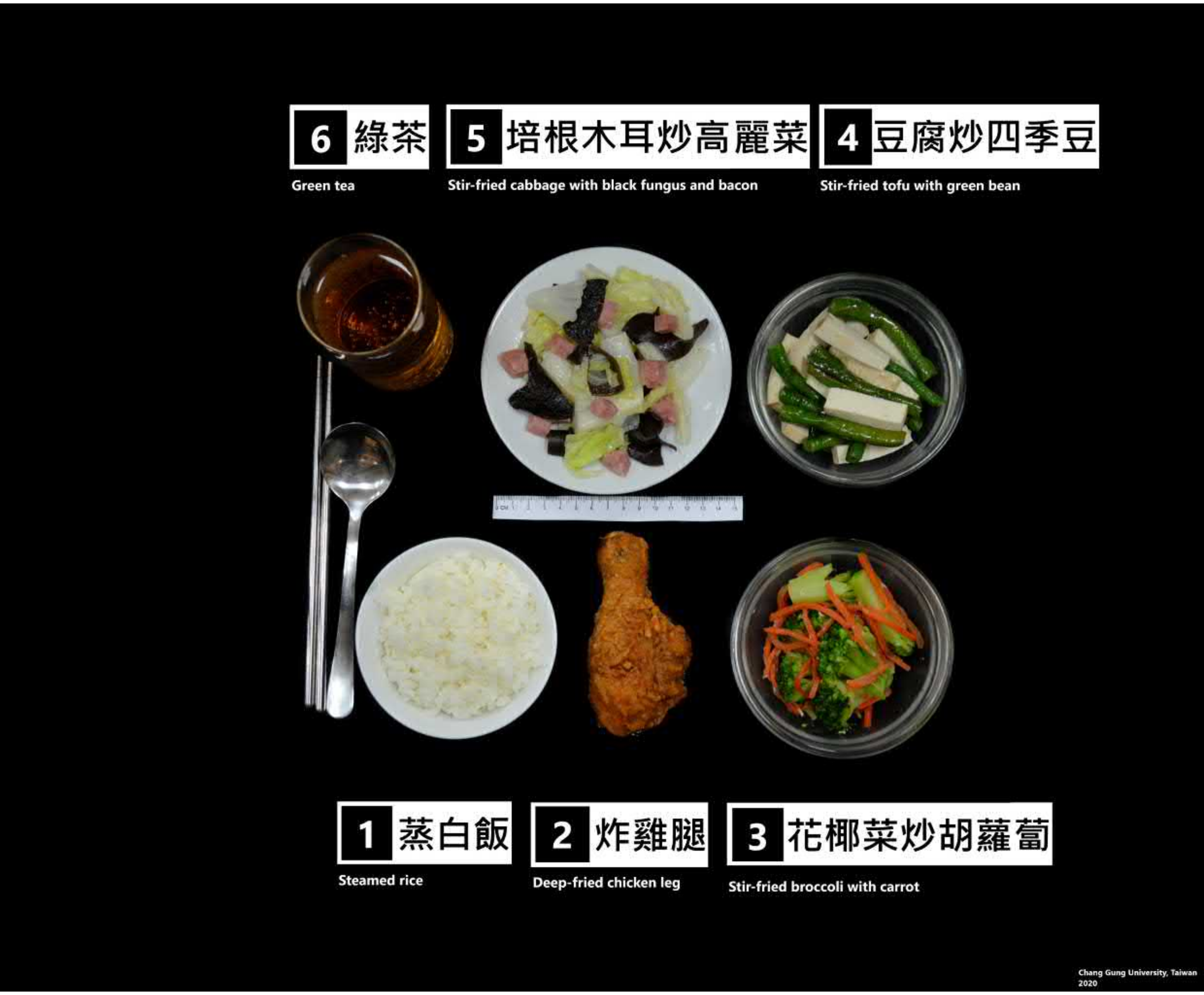
6 綠茶
Green tea
5 培根木耳炒高麗菜
Stir-fried cabbage with black fungus and bacon
4 豆腐炒四季豆
Stir-fried tofu with green bean
1 蒸白飯
Steamed rice
2 炸雞腿
Deep-fried chicken leg
3 花椰菜炒胡蘿蔔
Stir-fried broccoli with carrot
Chang Gung University, Taiwan
2020

Dinner Set

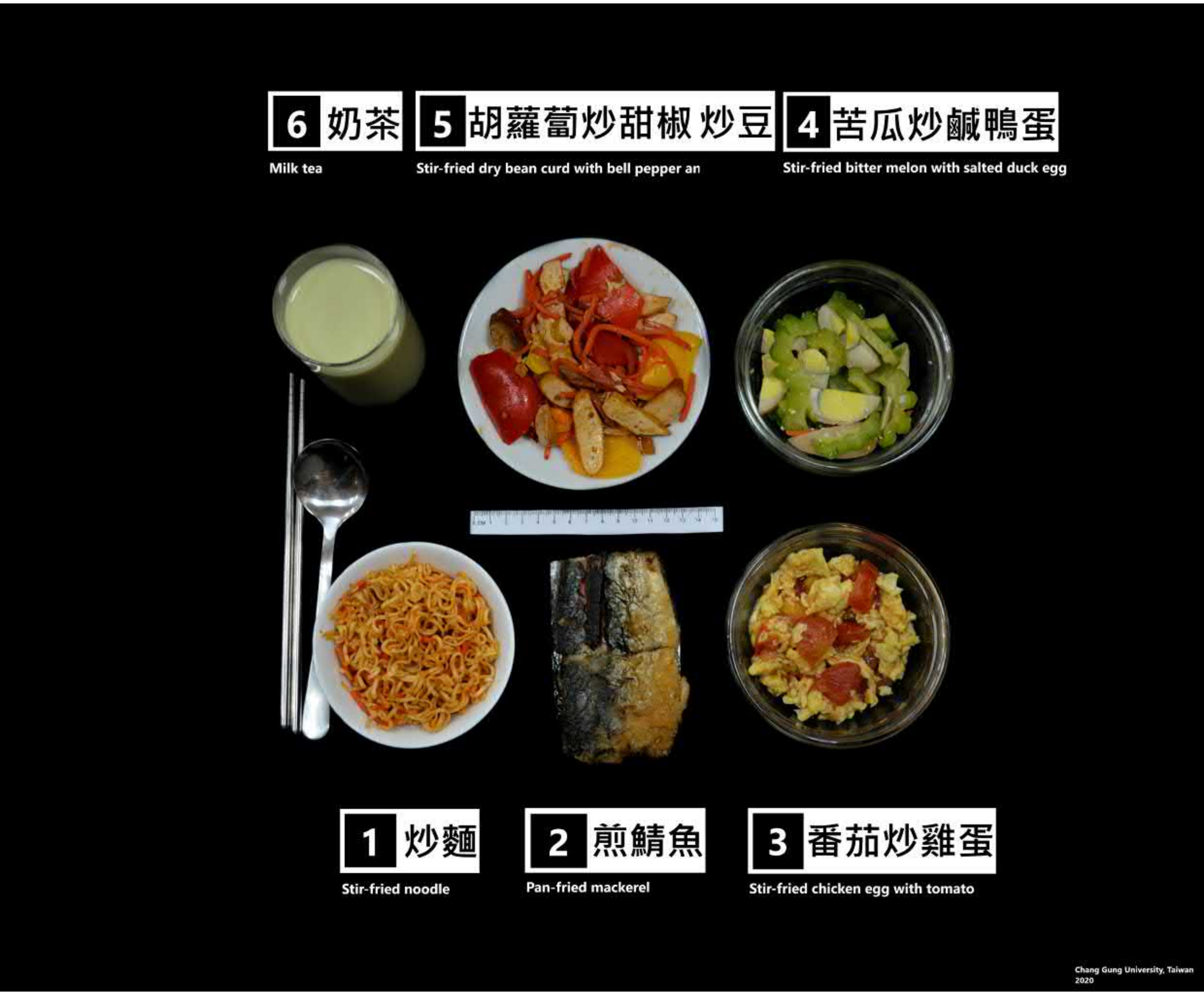

6 奶茶
Milk tea
5 胡蘿蔔炒甜椒 炒豆
Stir-fried dry bean curd with bell pepper an
4 苦瓜炒鹹鴨蛋
Stir-fried bitter melon with salted duck egg
1 炒麵
Stir-fried noodle
2 煎鯖魚
Pan-fried mackerel
3 番茄炒雞蛋
Stir-fried chicken egg with tomato
Chang Gung University, Taiwan
2020
