# Supplementary material for: Design and Usability Evaluation of Mobile Voice-Added Food Reporting for Elderly People: Randomized Controlled Trial
Source: JMIR Mhealth Uhealth. 2020 Sep 28;8(9):e20317. doi: 10.2196/20317 (PMC7551114; doi:10.2196/20317)
Supplement: Multimedia Appendix 6 [file mhealth_v8i9e20317_app6.docx]

Multimedia Appendix 6. Error types of dietary intake using a voice reporting approach.

| **Theme** | **Error type: Description** | **Example*** |
| --- | --- | --- |
| Trouble after voice reporting | (#1) Missing first food name/syllable(s): After verbal reporting, the presented answer list did not include the first food name or the first syllable(s) of the food names. | 1. An error was defined as missing the first food name, e.g., from “*tomato stir- fried chicken egg (fānqié-chǎo-jīdàn)”* to “*stir-fried chicken egg (chǎo-jīdàn)”*. The first food name, i.e., “*tomato (fānqié)”* was not presented*.* 2. An error was defined as missing the first food name syllable, e.g., from “*carrot (húluóbo)”* to “*rot (luóbo)”*. |
|  | (#2) Missing last food name/syllable(s): After verbal reporting, the presented answer list did not include the last food name or the last syllable(s) of the food names after voice reporting. | 1. An error was defined as missing the last food name, e.g., from “tofu stir-fried green bean (dòufu-chǎo-sìjìdòu)” to “tofu stir-fried (dòufu-chǎo)”*.* 2. An error was defined as missing the last food name syllable, e.g., from “wheat gluten (miàncháng)” to “wheat (miàn)”. |
|  | (#3) No desirable choices: After verbal reporting, the presented answer list did not present the desired food name or cooking method. | 1. Food name was recognized as another food name or words, e.g., from “rice (bái fàn)” to “white pea (bái wān)”, from “chicken egg (jīdàn)” to “eat egg (chīdàn)”, or from “pan-fried mackerel (zhēng yú)” to “steamed fish (zhēng yú)”. 2. Food attribute was recognized as another word, e.g., from “gravy (lǔ)” to “milk (rǔ)”, or from “stir-fried (chǎo)” to “grilled (kǎo)”, or from “pan-fried (jiān)” to “fried (jiān)”. 3. Participants’ incorrect foods reporting, e.g., from “pork sausage (zhūròu xiāngcháng)” to “pork chop (zhūpái)”, from “chicken leg (jītuǐ)” to “chicken steak (jīpái)”, or from “tofu (dòufu)” to “dried tofu (dòugān)”. |
|  | (#4) Missing cooking method(s): After verbal reporting, the presented answer list did not include the desired cooking method(s). | 1. In the VOR group, the participant did not verbally report the cooking method, e.g., from “boiled rice porridge (zhǔ xīfàn)” to “rice porridge (xīfàn)”. 2. In the VBR group, the participant did not include the “cooking method” button (see Fig. 3d-e and 4g-h). |
|  | (#5) Repeated pronunciations: The presented answer list showed repeated pronunciations of food names and/or food attributes after voice reporting. | 1. An error was defined as repeated food names, e.g., from “peanut gravy wheat gluten (huādòu-lǔ-miàncháng)” to “peanut gravy wheat gluten wheat gluten (huādòu-lǔ-miàncháng-miàn cháng)”. 2. An error was defined as repeated food attributes, e.g., from “peanut gravy wheat gluten (huādòu-lǔ-miàncháng)” to “peanut gravy gravy wheat gluten (huādòu-lǔ-lǔ-miàncháng)”. 3. An error was defined as repeated food name as well as food attribute, e.g., from “peanut gravy wheat gluten (huādòu-lǔ-miàncháng)” to “peanut gravy wheat...peanut gravy wheat gluten (huādòu-lǔ-miàn…-huādòu-lǔ-miàncháng)”. |
| Trouble in selecting one among the choices  (*click interaction*) | (#6) Incorrect selections in the list: Participant had trouble accurately tapping the desired choice (click interaction) leading to incorrect selection in the answer list. | 1. In the VOR group, the participant did not tap the desired choice (see step 2 in Fig. 1b and 2b for the correct selection). 2. In the VBR group, the participant made the wrong selection in the answer list. (see step 2 in Fig. 3b and 4b). 3. In the VBR group, the participant made the wrong selection in the list of possible food attributes (see step 4 in Fig. 3e and step 5 in Fig. 4h). 4. Participant should have clicked “cancel” but still tapped incorrect food names (see Fig. 1b, 2b, 3b, and 4b). 5. Participants should have not clicked “cancel” (see Fig. 1b, 2b, 3b, and 4b). |
| Trouble before dish completion  (*click interaction*) | (#7) Did not select the ‘mix’ button: Trouble before dish completion (click interaction). The user did not tap the “mix” button to complete dishes with two or three ingredients. | 1. The participant did not click the “mix” button for food combination step (see step 4 in Fig. 4f for the proper procedure). |
| Inappropriate user interaction | (#8) Incorrect operations: Incorrect operation procedure. | 1. The participant repeatedly clicked the voice button (see step 1 in Fig. 1a, 2a, 3a, and 4a for the proper procedure). 2. The participant did not select any food name (see step 2 in Fig. 1b, 2b, 3b, and 4b). 3. In the VOR group, the participant did not select any food names and continued to go for the next process (see step 2 in Fig. 1b and 2b for the proper procedure). 4. In the VBR group, the participant did not select food names among the five choices in the answer list and continue to go for the next process (see step 2 in Fig. 3b and 4b for the proper procedure). 5. In the VBR group, The participant repeatedly taped the food attribute button (see step 3 in Fig. 3d and 4d for the proper procedure). |

*: all words were direct translation
